# Continuous-Variable Quantum Key Distribution Based on Heralded Hybrid Linear Amplifier with a Local Local Oscillator

**DOI:** 10.3390/e23111395

**Published:** 2021-10-24

**Authors:** Yin Li, Yijun Wang, Yun Mao, Weishao Peng, Di Jin, Ying Guo

**Affiliations:** School of Automation, Central South University, Changsha 410083, China; liyin@csu.edu.cn (Y.L.); xxywyj@csu.edu.cn (Y.W.); yingguo@csu.edu.cn (Y.G.)

**Keywords:** continuous-variable quantum key distribution, optical amplifier, local local oscillator

## Abstract

An improved continuous variable quantum key distribution (CVQKD) approach based on a heralded hybrid linear amplifier (HLA) is proposed in this study, which includes an ideal deterministic linear amplifier and a probabilistic noiseless linear amplifier. The CVQKD, which is based on an amplifier, enhances the signal-to-noise ratio and provides for fine control between high gain and strong noise reduction. We focus on the impact of two types of optical amplifiers on system performance: phase sensitive amplifiers (PSA) and phase insensitive amplifiers (PIA). The results indicate that employing amplifiers, local local oscillation-based CVQKD systems can enhance key rates and communication distances. In addition, the PIA-based CVQKD system has a broader application than the PSA-based system.

## 1. Introduction

Quantum key distribution (QKD) provides a secret key sharing method guaranteed by quantum mechanics for trusted communication parties, namely Alice and Bob, in the presence of potential eavesdroppers [1,2,3]. Currently, there are two methods available for key distribution: discrete variable (DV) QKD [4,5] and continuous variable (CV) QKD [6,7,8,9]. Among them, the CVQKD has two main advantages. On the one hand, it circumvents the drawbacks of single-photon counting. On the other hand, it ensures that standard optical communication components are compatible [10,11]. The unconditional security of CVQKD has been established in the information-theoretical in both the asymptotic case [12,13] and the finite-size regime [14,15,16] to against general collective eavesdropping attacks.

The strong local oscillator (LO) for coherent detection as an important part of the CVQKD system can be employed as a filter to effectively suppress noise. However, the imperfections of the actual CVQKD system have led to potential loopholes and endangered the security of the communication system. Since Eve performs intercept-resend attacks by manipulating the LO, almost all of the attacks are related to the LO [17,18,19,20,21]. The CVQKD system based on local local oscillation (LLO), for example, prevents LO-related attacks by sending LO directly to the receiving end [22,23,24,25].

The transmission distance of CVQKD is currently limited compared to discrete variable systems, making it unsuitable for long-distance distribution. In the detection process, a heralded noiseless linear amplifier (NLA) is an excellent instrument for strengthening the amplitude of the coherent state while keeping the starting noise level low [26,27,28,29]. The practicality of this device has been proven in recent years, providing convincing evidence of the theory [30,31,32,33,34,35]. Furthermore, in Bob’s quadrature measurement, the defects associated with practical detectors cause a secret key rate constraint [36]. To compensate for this weakness, optical amplifier compensation technology offers a viable solution that can also improve transmission distance in specific situations [37,38,39].

We propose a CVQKD scheme based on LLO in this paper by placing an HLA at the detection end, which consists of a predictive measurement (MB)-based NLA and an optimal deterministic linear amplifier (DLA) that can amplify the amplitude of the coherent state while maintaining low noise and a high success rate. The signal-to-noise ratio is amplified by the NLA part, while the signal and success probability are amplified by the DLA part. In addition, we take into account the NLA’s restrictions. The addition of a hybrid amplifier to the LLO-based CVQKD protocol efficiently increases the key rate and the maximum secure transmission distance according to simulation results.

The paper is organized as follows. In Section 2, we describe the LLO-CVQKD system based on optical amplifiers. In Section 3, we conduct a theoretical analysis of excess noise. We performed a detailed analysis in Section 4, comparing the performance of practical systems that implement two different types of optical amplifiers. Finally, we conclude the paper in Section 5.

## 2. Description of the LLO-CVQKD Scheme Based on Optical Amplifiers

**Figure 1:**
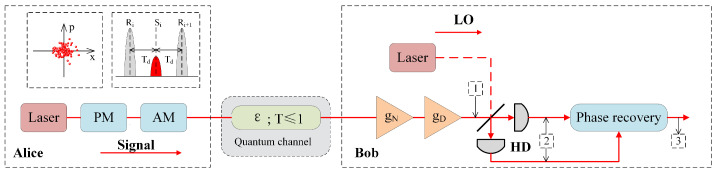
System layout of LLO-CVQKD scheme based on a heralded HLA. The signal light is in red (gray) solid line, and the local oscillator light is in red (gray) dashed line. LO, local oscillator; AM, amplitude modulator; PM, phase modulator; HD, heterodyne detection.

In this section, we introduce the LLO-CVQKD scheme by using heralded HLA. As shown in Figure 1, Alice randomly chooses two Gaussian random variables (with a mean of zero and a variance of $V_{A}$), generates a coherent state to modulate the continuous waves emitted by the laser [40,41]. An arbitrary phase rotation is generated by a quantum channel to change the states. To restore the original signal, a relatively strong phase reference pulse is alternately sent with the quantum signal. The beam then passes through the optical channel. At Bob’s side, a heralded HLA is placed in the signal beam to improve system performance. Besides, Bob performs optical heterodyne detection to measure quadrature *x* and *p*.

### 2.1. Amplifier Compensation Scheme

The above-mentioned heralded HLA is composed of a MB-NLA and an optimal DLA. In theory, NLA can increase the amplitude of the coherent state while keeping the original noise level constant [26]. The output after an initial channel and NLA action, given any quantum state ${\hat{\rho }}_{in}=\int d^{2}\gamma P_{in}\left(\gamma \right)\left|\gamma \rangle \langle \gamma \right|$, can be represented as

(1) $$\begin{array}{c} {\hat{\rho }}_{out}^{NLA}\propto \int d^{2}\gamma P_{in}\left(\gamma \right)\hat{\sigma }\left(\gamma \right)e^{{\left|\gamma \right|}^{2}T\frac{\left(g_{N}^{2}-1\right)\left(1-\lambda_{ch}^{2}\right)}{1-g_{N}^{2}\lambda_{ch}^{2}}}, \end{array}$$

where $g_{N}$ describes the operator of NLA. The initial channel transforms the coherent state of the mean amplitude γ into the thermal state of the parameter $\lambda_{ch}$ and the average amplitude $\sqrt{T}\gamma$. The displaced thermal state can be expressed by

(2) $$\begin{array}{c} \hat{\sigma }\left(\gamma \right)=\hat{D}\left(\tilde{g_{N}}\sqrt{T}\gamma \right){\hat{\rho }}_{th}\left(g_{N}\lambda_{ch}\right){\hat{D}}^{†}\left(\tilde{g_{N}}\sqrt{T}\gamma \right), \end{array}$$

of parameter $g\lambda_{ch}$ and mean amplitude $\tilde{g_{N}}\sqrt{T}\gamma$, where the gain $\tilde{g}$ is given by

(3) $$\begin{array}{c} \tilde{g_{N}}=g_{N}\frac{1-\lambda_{ch}^{2}}{1-g_{N}^{2}\lambda_{ch}^{2}}. \end{array}$$

**Figure 2:**
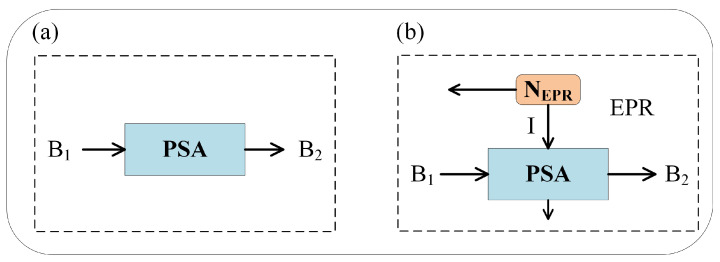
Two different amplifier model. (**a**) Model for a phase-sensitive amplifier. (**b**) Model for a phase-insensitive amplifier.

We mainly consider two modes of DLA, phase-sensitive amplifiers (PSA) and phase-insensitive amplifiers (PIA) [42,43,44,45]. PSA ideally allows noiseless amplification of a selected quadrature. The structure of PSA is shown in Figure 2a. $X_{B}^{i}$ and $P_{B}^{i}$ correspond to the outputs at point i in Figure 1. The output of the amplifier is written as

(4) $$\left[\begin{array}{c} X_{B}^{0}\\ P_{B}^{0} \end{array}\right]=\left[\begin{array}{cc} \sqrt{g_{D}} & 0\\ 0 & 1/\sqrt{g_{D}} \end{array}\right]\left[\begin{array}{c} X_{A}\\ P_{A} \end{array}\right]$$

where $g_{D}$ is the amplifier coefficient of DLA. Besides, the noise introduced by the real amplifier is ignored here.

PIA is a non-degenerate optical parametric amplifier whose structure is shown in Figure 2b. It consists of a noiseless amplifier and a two-mode squeezed vacuum (EPR) state of variance $N_{EPR}$, which is used to simulate the inherent noise of the amplifier. The output of PIA is written as

(5) $$\left[\begin{array}{c} X_{B}^{0}\\ P_{B}^{0} \end{array}\right]=\left[\begin{array}{cc} X_{A} & X_{I}\\ P_{A} & P_{I} \end{array}\right]\left[\begin{array}{c} \sqrt{g_{D}}\\ \sqrt{g_{D}-1} \end{array}\right],$$

where *I* is an idler mode that is ideally in a vacuum state or in a state featuring a noise variance $V_{I}=N_{EPR}>1$.

### 2.2. Phase Recovery Scheme

Bob’s measurement results are described by

(6) $$\left[\begin{array}{c} X_{B}^{1}\\ P_{B}^{1} \end{array}\right]=\left[\begin{array}{cc} cos\varphi & sin\varphi \\ -sin\varphi & cos\varphi \end{array}\right]\left[\begin{array}{c} X_{B}^{0}\\ P_{B}^{0} \end{array}\right]+\left[\begin{array}{c} N_{X}\\ N_{P} \end{array}\right],$$

where φ respects the phase drift in a quantum channel. $N_{X}$ and $N_{P}$ are independent and identically distributed (i.i.d.) Gaussian noises.

Assuming that the channel drift is known, Bob’s measurement can be corrected as

(7) $$\left[\begin{array}{c} X_{B}^{2}\\ P_{B}^{2} \end{array}\right]=\left[\begin{array}{cc} cos\varphi & -sin\varphi \\ sin\varphi & cos\varphi \end{array}\right]\left[\begin{array}{c} X_{B}^{1}\\ P_{B}^{1} \end{array}\right],$$

Based on PSA scheme, the final output can be obtained according to Equations (4), (6) and (7), which can easy to show

(8) $$\left[\begin{array}{c} X_{B}^{2}\\ P_{B}^{2} \end{array}\right]=\left[\begin{array}{cc} \sqrt{g_{D}} & 0\\ 0 & 1/\sqrt{g_{D}} \end{array}\right]\left[\begin{array}{c} X_{A}\\ P_{A} \end{array}\right]+\left[\begin{array}{c} N_{X}^{\prime }\\ N_{P}^{\prime } \end{array}\right],$$

where noise terms can be expressed as

(9) $$\left[\begin{array}{c} N_{X}^{\prime }\\ N_{P}^{\prime } \end{array}\right]=\left[\begin{array}{cc} cos\varphi & -sin\varphi \\ sin\varphi & cos\varphi \end{array}\right]\left[\begin{array}{c} N_{X}\\ N_{P} \end{array}\right].$$

Based on PIA scheme, the final output can be obtained according to Equations (5)–(7), which can easily show

(10) $$\left[\begin{array}{c} X_{B}^{2}\\ P_{B}^{2} \end{array}\right]=\sqrt{g_{D}}\left[\begin{array}{c} X_{A}\\ P_{A} \end{array}\right]+\left[\begin{array}{c} N_{X}^{\prime }\\ N_{P}^{\prime } \end{array}\right],$$

where noise terms can be described by

(11) $$\left[\begin{array}{c} N_{X}^{\prime }\\ N_{P}^{\prime } \end{array}\right]=\left[\begin{array}{cc} cos\varphi & -sin\varphi \\ sin\varphi & cos\varphi \end{array}\right]\left[\begin{array}{c} N_{X}\\ N_{P} \end{array}\right]+\sqrt{g_{D}-1}\left[\begin{array}{c} X_{I}\\ P_{I} \end{array}\right].$$

Next, calculate the phase shift. Assume that the phase drift is slow enough to remain constant over the frame time of ΔT. Since Alice alternately sends the quantum signal and the reference pulse, the phase shift of the signal pulse can be calculated from the phase shift of the reference pulse [46]. The phase of the reference pulse can be expressed as

(12) $$\begin{array}{c} \varphi_{R}^{i}=-tan^{-1}\frac{P_{R}^{i}}{X_{R}^{i}}. \end{array}$$

The phase shift of the signal pulse can be calculated as

(13) $$\begin{array}{c} \varphi_{S}^{i}=\frac{\varphi_{R}^{i}+\varphi_{R}^{i+1}}{2}=\varphi_{R}^{i}+2\pi f_{d}T_{d}, \end{array}$$

where $f_{i}=\left(\varphi_{R}^{i+1}-\varphi_{R}^{i}/4\pi T_{d}\right)$ is the frequency difference between two adjacent reference pulsed lasers in a short time.

## 3. Excess Noise Analysis

The excess noise analysis of the LLO CV-QKD system based on the heralded HLA is shown in this section. Although the signal’s pulse phase was approximated using the reference pulse phase in the preceding section, it was not exact enough, and there were inaccuracies. In the phase recovery phase, the excess noise induced by the uncertainty of φ can be described as

(14) $$\begin{array}{c} \varepsilon_{\varphi }=V_{A}\sigma_{\varphi }, \end{array}$$

where $\sigma_{\varphi }$ is the variance of noise generated by the uncertainty of phase φ. The noise variance $\sigma_{\varphi }$ is expressed as

(15) $$\begin{array}{c} \sigma_{\varphi }=\frac{1}{2}\left\{\langle {\left(\Delta \varphi_{S}T_{d}\right)}^{2}\rangle +\langle {\left(\Delta \varphi_{L}T_{d}\right)}^{2}\rangle \right\}, \end{array}$$

where $\langle {\left(\Delta \varphi_{S}T_{d}\right)}^{2}\rangle$ and $\langle {\left(\Delta \varphi_{L}T_{d}\right)}^{2}\rangle$ are the phase noise of signal light and auxiliary light, respectively. $T_{d}$ represents the time interval between the signal pulse and the reference pulse (see Figure 1). Assuming that the laser phase at t=0 is $\varphi_{0}$, the phase noise φ(t) can be modeled as a Gaussian random variable with a mean of 0 and a variance of

(16) $$\begin{array}{c} \langle {\left(\Delta \varphi_{S}t\right)}^{2}\rangle =\frac{2t}{\alpha }, \end{array}$$

where $\alpha ⋍\frac{1}{\pi \Delta f}$ respects the coherence time of the laser. Δf is the linewidth of α.

## 4. Security Analysis

This section will analyze the impact of the amplifier on the performance of the LLO-CVQKD system. Our simulation is based on the security of GMCS QKD system given in Refs. [14,47].

An NLA-based CV-QKD system with a variance $V\left(\delta \right)=\frac{1+\delta^{2}}{1-\delta^{2}}$, channel transmittance $T=10^{-\alpha L/10}$, and excess noise ε can be transformed into another typical CV-QKD system with variance $V\left(\delta_{N}\right)=\frac{1+\delta_{N}^{2}}{1-\delta_{N}^{2}}$, channel transmittance $T_{N}$, excess noise $\varepsilon_{N}$, and loss coefficient α = 0.2 dBkm ${}^{-1}$. The parameter expression that has been equivalently converted is as follows:

(17) $$\begin{array}{c} \delta_{N}=\delta \sqrt{\frac{\left(g_{N}^{2}-1\right)\left(\varepsilon -2\right)T-2}{(g_{N}^{2}-1)\varepsilon T-2}},\\ T_{N}=\frac{g^{2}T}{\left(g_{N}^{2}-1\right)T\left[\frac{1}{4}\left(g_{N}^{2}-1\right)\left(\varepsilon -2\right)\varepsilon T-\varepsilon +1\right]+1},\\ \varepsilon_{N}=\varepsilon -\frac{1}{2}\left(g^{2}-1\right)\left(\varepsilon -2\right)\varepsilon T. \end{array}$$

When the parameters in the CVQKD system above satisfy a specified condition, the system can be viewed as equivalent. The limitations are written as

(18) $$\begin{array}{c} 0\leq \delta_{N}<1\Rightarrow 0\leq \delta <{\left(\sqrt{\frac{\left(g_{N}^{2}-1\right)\left(\varepsilon -2\right)T-2}{(g_{N}^{2}-1)\varepsilon T-2}}\right)}^{-1}. \end{array}$$

**Figure 3:**
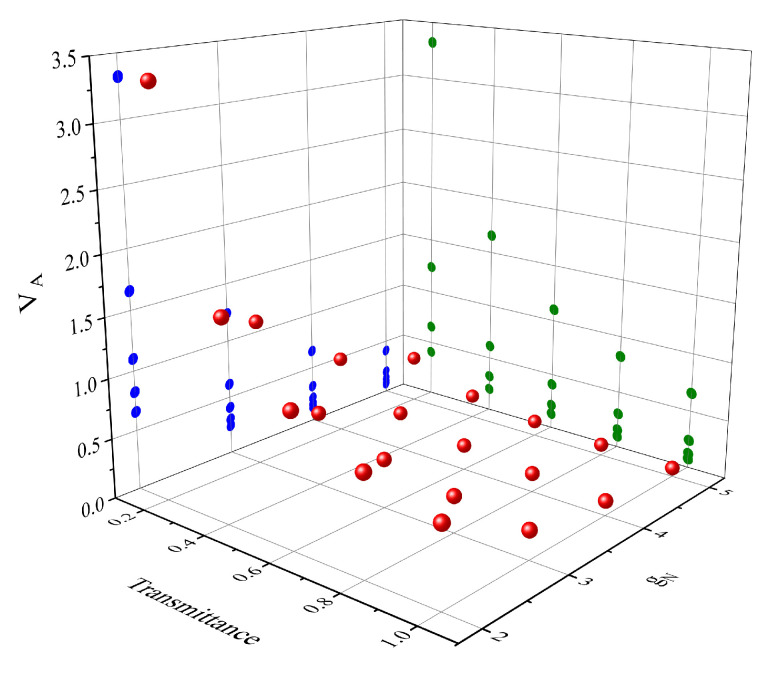
Maximum value of the modulation variance. Five cases at different transmittance (from T=0.2 to 1 in steps of 0.2) and four amplification factor of NLA (from $g_{N}=2$ to 5 in steps of 1) are investigated.

Figure 3 shows the limitations of VA taking under various settings when the excess noise is equal to 0.01. In addition the success probability of the amplifier is taken into account. According to Ref. [27], the upper bound on the amplifier success probability is

(19) $$\begin{array}{c} P_{success}\leq \frac{1}{g_{N}^{2}}. \end{array}$$

The secure key rate with finite-size effect is given by

(20) $$\begin{array}{c} R=\frac{nP_{success}}{N}\left[\beta I_{AB}-\chi_{BE}-\Delta \left(n\right)\right], \end{array}$$

where $I_{AB}$ is defined as the Shannon mutual information of Alice and Bob, $\chi_{BE}$ respects the upper bound of the Holevo information between Eve and Bob, Δ(n) is related to the security of the privacy amplification and β is the reconciliation efficiency. The privacy amplification Δ(n) is given by

(21) $$\begin{array}{c} \Delta \left(n\right)\equiv \left(2dimH_{X}+3\right)\sqrt{\frac{log_{2}\left(2/\bar{\epsilon }\right)}{n}}+\frac{2}{n}\left(1+\epsilon_{PA}\right), \end{array}$$

where $\bar{\epsilon }$ and $\epsilon_{PA}$ are the smoothing parameter and failure probability of the privacy amplification, respectively. $H_{X}$ is the Hilbert space corresponding to the variable *X* used in the raw key. The square-root term in Δ(n) actually corresponds to the pace with which the smooth min-entropy of an independently and identically distributed (i.i.d.) state approaches the von Neumann entropy. Indeed, the smooth min-entropy of an i.i.d. state is identical to its von Neumann entropy only at the asymptotic limit. The second term is proportional to the privacy amplification procedure’s failure probability.

The Shannon mutual information of Alice and Bob, as well as the Holevo information of Eve and Bob, differ depending on the amplifier type. The CVQKD scheme based on PSA is first studied. The detection-added noise of quadrature X and P is considered separately, as shown below

(22) $$\begin{array}{c} \chi_{PSA}^{x}=\frac{1+\left(1+\eta \right)+2\vartheta_{el}}{g_{D}\eta },\\ \chi_{PSA}^{p}=\frac{g_{D}\left(1+\left(1+\eta \right)+2\vartheta_{el}\right)}{\eta }, \end{array}$$

where $\vartheta_{el}$ is the detector electronics noise, η is detector efficiency. The total noise is defined as

(23) $$\begin{array}{c} \chi_{tot}^{x,p}=\chi_{line}+\chi_{PSA}^{x,p}, \end{array}$$

where $\chi_{line}=1/T_{N}-1+\varepsilon_{N}$ is the total channel-added noise. The Shannon mutual information of Alice and Bob is written by [48]

(24) $$\begin{array}{c} I_{AB}=log_{2}\frac{V_{B}}{V_{B|A}}, \end{array}$$

where $V_{B}=\frac{\eta T_{N}}{2}{\left[\left(V_{N}+\chi_{tot}^{x}\right)\left(V_{N}+\chi_{tot}^{p}\right)\right]}^{1/2}$, and $V_{B|A}=\frac{\eta T_{N}}{2}{\left[\left(1+\chi_{tot}^{x}\right)\left(1+\chi_{tot}^{p}\right)\right]}^{1/2}$. The Holevo information between Eve and Bob is defined as [12,13]

(25) $$\begin{array}{c} \chi_{BE}=\sum_{i=1}^{2}G\left(\frac{\lambda_{i}-1}{2}\right)-\sum_{i=3}^{5}G\left(\frac{\lambda_{i}-1}{2}\right), \end{array}$$

where $G\left(x\right)=\left(x+1\right)log_{2}\left(x+1\right)-xlog_{2}x$. The symplectic eigenvalues $\lambda_{1,2}$ are given by

(26) $$\begin{array}{c} \lambda_{1,2}=\frac{1}{2}\left[A\pm \sqrt{A^{2}-4B}\right], \end{array}$$

where $A=V_{N}^{2}\left(1-2T_{N}\right)+2T_{N}+T_{N}^{2}{\left(V_{N}+\chi_{line}\right)}^{2}$ and $B=T_{N}^{2}{\left(V\chi_{line}+1\right)}^{2}$. The symplectic eigenvalues $\lambda_{3,4}$ are expressed as

(27) $$\begin{array}{c} \lambda_{3,4}=\frac{1}{2}\left[C\pm \sqrt{C^{2}-4D}\right], \end{array}$$

where

(28) $$\begin{array}{c} C_{PAS}=\frac{A\chi_{PSA}^{x}\chi_{PSA}^{p}+B+1+\left(\chi_{PSA}^{x}+\chi_{PSA}^{p}\right)\left(V_{N}\sqrt{B}+T_{N}\left(V_{N}+\chi_{line}\right)\right)+2T_{N}\left(V_{N}^{2}-1\right)}{\left(T_{N}\left(V_{N}+\chi_{tot}^{x}\right)\right)\left(T_{N}\left(V_{N}+\chi_{tot}^{p}\right)\right)}, \end{array}$$

and

(29) $$\begin{array}{c} D_{PSA}=\left(\frac{V_{N}+\sqrt{B}\chi_{PSA}^{x}}{T_{N}\left(V_{N}+\chi_{tot}^{x}\right)}\right)\left(\frac{V_{N}+\sqrt{B}\chi_{PSA}^{p}}{T(V_{N}+\chi_{tot}^{p})}\right). \end{array}$$

The last eigenvalue is $\lambda_{5}=1$.

Then, the LLO-CVQKD scheme based on PIA is analyzed. The detection-added noise is shown as

(30) $$\begin{array}{c} \chi_{PIA}=\frac{1+\left(1-\eta \right)+2\vartheta_{el}+N_{EPR}\left(g-1\right)\eta }{g\eta }, \end{array}$$

where $N_{EPR}$ is the variance of the EPR state to model the amplifier’s inherent noise. A similar change was made to the Shannon information. We now have

(31) $$\begin{array}{c} V_{B}=\eta T_{N}\left(V+\chi_{tot}\right)/2,\\ V_{B|A}=\eta T_{N}\left(1+\chi_{tot}\right)/2. \end{array}$$

$\chi_{BE}$ is calculated from

(32) $$\begin{array}{c} \chi_{BE}=\sum_{i=1}^{2}G\left(\frac{\lambda_{i}-1}{2}\right)-\sum_{i=3}^{7}G\left(\frac{\lambda_{i}-1}{2}\right), \end{array}$$

The parameters ($\lambda_{1,2,3,4}$) are calculated according to Equations (26) and (27), while $\lambda_{5,6,7}=1$. The parameters ($\lambda_{3,4}$) are calculated from

(33) $$\begin{array}{c} C_{PIA}=\frac{A\chi_{PIA}^{2}+B+1+2\chi_{PIA}\left(V_{N}\sqrt{B}+T_{N}\left(V_{N}+\chi_{line}\right)\right)+2T_{N}\left(V_{N}^{2}-1\right)}{{\left(T_{N}\left(V_{N}+\chi_{tot}\right)\right)}^{2}}, \end{array}$$

and

(34) $$\begin{array}{c} D_{PIA}={\left(\frac{V_{N}+\sqrt{B}\chi_{PIA}}{T_{N}\left(V_{N}+\chi_{tot}\right)}\right)}^{2}. \end{array}$$

**Figure 4:**
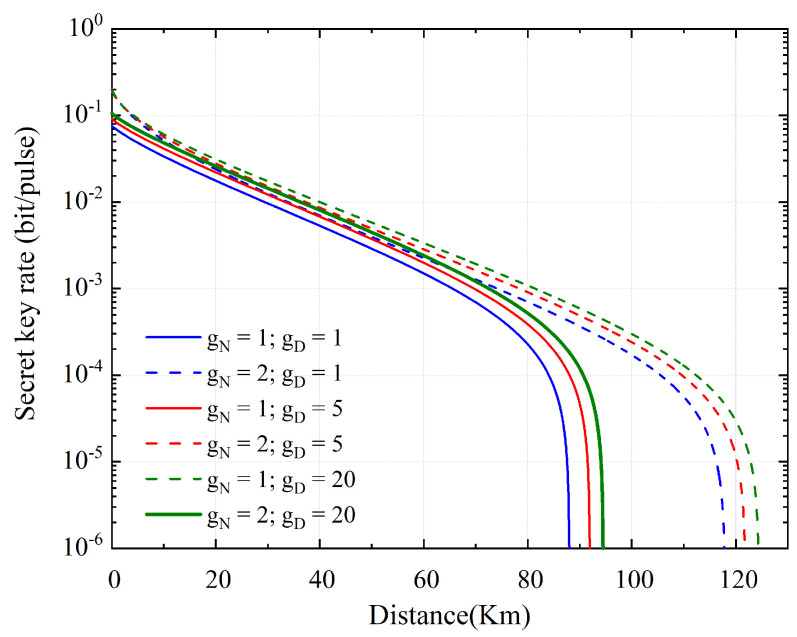
The secret key rate as a function of transmission distance based on PSA. Blue line, red line, and green line correspond, respectively, to a value of the amplifier coefficient of $g_{D}=1$ (initial), $g_{D}$ = 5, and $g_{D}$ = 20. Full lines and dashed lines correspond to two cases where the number of $g_{N}$ links is 1 and 2, respectively.

The secret key rate as a function of transmission distance is shown in Figure 4 using HLA with PSA. For simulation, the parameters stated in Table 1 are utilized, which is in accordance with the most advanced experimental methods [22,24]. The secure communication distance increases as the amplifier coefficient $g_{N}$ and $g_{D}$ increases when the modulation variance $V_{A}=0.6$. In terms of both key rate and distance, NLA-based schemes perform better than PSA-based systems. When the solution is based on HLA, the performance is optimal.

**Figure 5:**
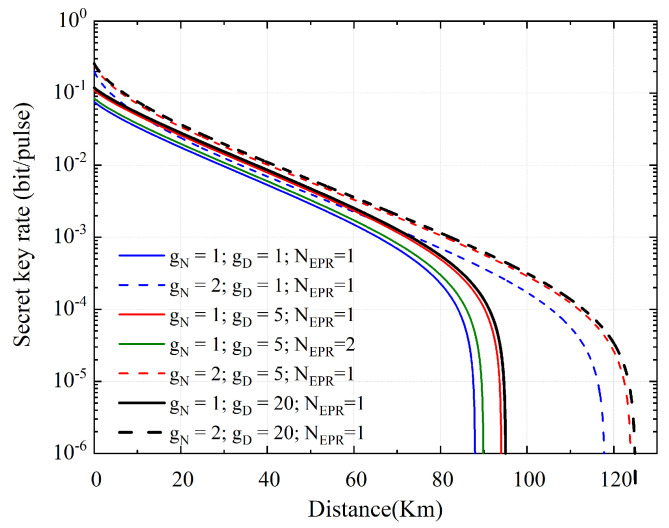
The secret key rate as a function of transmission distance based on PIA. Blue line, red line, and black line correspond, respectively, to a value of the amplifier coefficient of $g_{D}=1$ (initial), $g_{D}=5$, and $g_{D}=20$. The green line represents the case of $N_{EPR}=2$. Full lines and dashed lines correspond to two cases where the number of $g_{N}$ links is 1 and 2, respectively.

The secret key rate as a function of transmission distance is plotted in Figure 5 using HLA with PIA. The PIA-based LLO-CVQKD is similar to the psa-based LLO-CVQKD. The system performance improves as the amplification parameters are increased. When $N_{EPR}$ is increased, however, the system performance degrades due to an increase in the introduced noise.

## 5. Conclusions

In this study, we offer a practical LLO-CVQKD system based on an HLA, which consists of an MB-NLA and a DLA, with the latter compensating for the former’s signal-to-noise ratio degradation. Two HLA schemes based on two separate DLAs, a phase-sensitive amplifier and a phase-insensitive amplifier, were analyzed to verify system performance. The system’s noise was initially studied, with all conceivable consequences taken into account. Following that, simulations were run to determine the situations under which the NLA is applicable. Finally, the system’s performance was assessed utilizing two alternative amplifier methods. The results demonstrated that HLAs based on various forms of DLA can help improve system performance. Furthermore, when compared to the PSA-based scheme, the PIA-based strategy performs better. Because the majority of the simulation parameters were derived from existing experiments, our research was extremely practical.

In addition, we supplemented the scheme in Refs. [28,29]. Ref. [28] proposes a basic CVQKD scheme for Gaussian modulated coherent states based on hybrid amplifiers. Ref. [29] proposes a CVQKD scheme based on a four-state modulation scheme using a hybrid amplifier under seawater channel conditions. Our scheme is mainly based on the improvement of Ref. [28]. We mainly analyze the performance of the HLA scheme in the case of local local oscillation. Furthermore, the effective case of HLA is analyzed.

## Figures and Tables

**Table 1:** The parameters to simulate the secret key rate.

Parameter	Value	Description
$V_{A}$	0.6	Modulation variance
β	98%	Reconciliation efficiency
η	0.6	Detector efficiency
$\nu_{el}$	0.05	Detector noise
$\sigma_{\varphi }$	0.04	Phase noise variance
$\bar{\epsilon },\epsilon_{PA}$	$10^{-20}$	Security parameter
N	$10^{10}$	Total photons
$\frac{n}{N}$	$\frac{1}{2}$	Photon ratio used for key

## Data Availability

The data used are included in the article.
